# Patterns of suicide deaths in Hungary between 1995 and 2017

**DOI:** 10.1016/j.ssmph.2021.100958

**Published:** 2021-11-07

**Authors:** Tamás Lantos, Richard J.Q. McNally, Tibor András Nyári

**Affiliations:** a Department of Medical Physics and Informatics, Faculty of Medicine, University of Szeged, 9 Korányi Alley, 6720, Szeged, Hungary; b Population Health Sciences Institute, Faculty of Medical Sciences, Newcastle University, Royal Victoria Infirmary, Queen Victoria Road, Newcastle Upon Tyne, NE1 4LP, England, United Kingdom

**Keywords:** Suicide mortality, Risk factors, Seasonal variation, Suicide methods, Epidemiology, Hungary

## Abstract

Hungary has had one of the highest suicide mortality rates in the world for decades. Investigating seasonality of suicide deaths is essential as its findings could be key elements in the prevention. In our study we have analyzed the seasonal effect in suicide mortality in relation to possible risk factors in Hungary during 1995–2017.

Data on the numbers of suicide deaths were obtained from a published online database. Negative binomial regression was employed to investigate the effect of possible risk factors and seasonal and annual trends in suicide rates. The seasonal effect was further investigated, adding a significant risk factor from the “initial” negative binomial regression.

The suicide risk was significantly (*p* < 0.001) higher in men than in women (incidence rate ratio: 3.48), and it increased with age and decreased with education level. Marriage was a protective factor against suicide. Annual suicide mortality declined significantly (*p* < 0.001 for trend) from 36.7 (95% confidence interval: 35.5–37.9) to 16.5 (15.7–17.3) per 100,000 persons per year during the study period. Significant seasonality was found in suicide rates with a peak in late June. Similar peaks were observed at each level of each risk factor. There were differences in peaks by suicide method. The peak of non-violent suicides was in early June; suicides committed by violent methods peaked half a month later.

This study suggests that there was a significant seasonal effect on suicide deaths between 1995 and 2017, which remained significant even in the presence of each risk factor. To our knowledge, this has been the first study to investigate the seasonal pattern so extensively in Hungary. Our findings confirm that the environmental effects are involved in the etiology of suicide mortality.

## Highlights

• Significant seasonality was found in suicide rates with a peak in late June.
• Seasonal effect remained almost unchanged after adjusting sociodemographic factors.
• There were differences in seasonal peaks by suicide method.
• Environmental effects are involved in the etiology of suicide mortality.

## 1 Introduction

From 1960, the Hungarian suicide mortality rate had been consistently among the highest ones for decades (Rihmer et al., 2013). During the 1995–2017 period, Hungary recorded the third highest standardized suicide rate (on average) from among the 27 members of the European Union (only behind Lithuania and Latvia, and narrowly followed by Estonia) and by far the highest among the Visegrad Group (V4) countries (the Czech Republic, Hungary, Poland and Slovakia) (World Health Organization, 2018). Suicide was the leading external cause of death in Hungary in this period with more than 2600 deaths per year on average (Hungarian Central Statistical Office, 2019).

Investigating seasonality of suicide deaths is essential as its findings could be key elements in the prevention. According to an overview (Christodoulou et al., 2012), “The seasonal variation is evident in Eastern European countries.” In addition, despite the weakening seasonal link in Hungary (Lester & Moksony, 2003), seasonality remained very strong compared to other countries (Voracek et al., 2004). Previous Hungarian studies (Sebestyen et al., 2010; Zonda, 2005) have reported a seasonal peak in early summer (May–June). Similar peaks were detected in other Central European countries, such as Austria (for June) and the Czech Republic, Germany and Switzerland (all for May) (Petridou et al., 2002). This can partly be explained by the “relative unhappiness” phenomenon (due to increased intensity of social life (Preti, 2002)) and the “broken-promise effect” proposed by Gabennesch (Gabennesch, 1988) (with the beginning of summer, unfulfilled expectations lead to extreme forms of disappointment).

Several risk factors for suicide are already known, including sociodemographic factors, such as gender (Hawton, 2000), age (Stack, 2000) and marital status (Kposowa, 2000) (i.e., males, older people and non-married people are more likely to take their own lives). Moreover, educational attainment shows one of the most consistent and strongest relationships with various indicators of health status (Elo et al., 2009) (including those that might lead to suicidal ideation). Furthermore, apart from these factors, the role of suicide method might also be important in a seasonal investigation of suicide mortality (as seasonal peaks could vary by suicide method).

In our study, the annual trends (in general) and seasonal patterns (adjusted to one of the potential risk factors: gender, age, region, marital status and educational attainment, respectively) for suicide rates were investigated in Hungary during the 23-year period between January 1, 1995 and December 31, 2017 (as this had been the longest accessible interval at the start of the study).

## 2 Methods

### 2.1 Study population and source of data

The 23-year period between 1995 and 2017 was considered in this analysis. There were no monthly population data, therefore we have used annual population data and the monthly birth and death data to estimate the numbers of population by months. Data on yearly population were obtained from a published nationwide population register operated by the *Hungarian Central Statistical Office* (HCSO) (Hungarian Central Statistical Office, 1996–2018).

The HCSO provides data on the number of births for each month over the study period but by gender only for each year. The number of births in each month for each gender was estimated assuming no monthly variation in the gender ratio within any year.

The monthly number of deaths are published online (*Dissemination Database*) by the HCSO (Hungarian Central Statistical Office, 2019). Thus, monthly population estimates were calculated based on the monthly number of births and deaths.

The data on suicide deaths were also available on the online HCSO database (Hungarian Central Statistical Office, 2019). Some cells contained protected data. We considered these ‘missing’ fields to be 1 (as in vast majority of cases, this must be the true value). This can cause some discrepancies in marginal numbers, but these differences are negligible.

The classification of suicide methods was based on the International Classification of Diseases (ICD; 9th Revision [prior to 1996]: E950-E959; 10th Revision [since 1996]: X60-X84, Y87). Based on these codes, the following *suicide methods* were distinguished: (1) “Hanging” (1995: E953/1996–2017: X70), (2) “Self-poisoning by drugs” (E950.0-E950.5/X60–64), (3) “Self-poisoning by chemicals and other substances” (E950.6-E950.9/X65–66, X68–69), (4) “Self-poisoning by gases” (E951-E952/X67), (5) “Jumping from a height” (E957/X80), (6) “Drowning” (E954/X71), (7) “Firearm” (E955.0-E955.4/X72–74) and (8) “Other/unspecified” (all remaining codes). These (specified) methods were grouped into two main categories: violent methods (subcategories 1, 5, 6 and 7) and non-violent ones (2, 3 and 4).

**Fig. 1:**
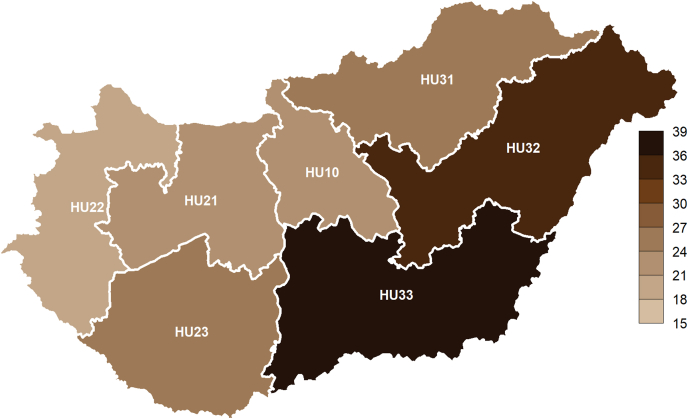
NUTS2 regions of Hungary colored by crude suicide rates (number of deaths per 100,000 population) during 1995–2017. Notes: *NUTS2* Nomenclature of territorial units for statistics (from the French version Nomenclature des Unités territoriales statistiques), 2nd level; Codes: *HU10* Central Hungary, *HU21* Central Transdanubia, *HU22* Western Transdanubia, *HU23* Southern Transdanubia, *HU31* Northern Hungary, *HU32* Northern Great Plain, *HU33* Southern Great Plain.

Territorial units were based on the second level of NUTS 2013 (*Nomenclature des unités territoriales statistiques* – Nomenclature of Territorial Units for Statistics, 2013 revision) classification (Eurostat, 2015). These *statistical regions* (second level of the classification; NUTS2) were as follows (Fig. 1): Central Hungary (HU10), Central Transdanubia (HU21), Western Transdanubia (HU22), Southern Transdanubia (HU23), Northern Hungary (HU31), Northern Great Plain (HU32) and Southern Great Plain (HU33).

*Marital status* was used as a binary variable (married vs. non-married) in the analyses. The whole population was considered first, then, as a “more relevant” subgroup (in this respect), only the over-20-year-old age group was taken into consideration. It is worth mentioning that children under 16 may only marry with the permission of child protective services in Hungary (pursuant to Act V of 2013 on the Civil Code, Section 4(9) [Legal age for marriage]). Although the legal aspect of a relationship is not the only one to affects someone's life, other categorization (e.g. single vs. non-single) were not possible as there are no such population-level data to investigate suicide deaths according to these categories.

*Educational attainment* was classified according to the ISCED-97 (International Standard Classification of Education, 1997 version) system (Hungarian Institute for Educational Research and Development, 2007; European Centre for the Development of Vocational Training, 2011): less than eight years of primary school (ISCED level 0–1A), eight years of primary school (2A), vocational qualification without secondary school-leaving certificate (vocational schools, 2C–3C), secondary school-leaving certificate (secondary vocational schools and grammar schools, 3A) and higher education degree (colleges and universities, 5A). In line with the above, population data were only available from 1998 (and only for the 15–74-year-old age group).

Data on both the population and number of deaths due to suicide were classified by *age group* as follows: 0–19 years (“youth”), 20–34 years (“young adults”), 35–49 years (“middle-aged adults”), 50–64 years (“older adults”) and over 65 years (“elderly”/pensioners).

For the general population, age-specific death rates were calculated. The suicide mortality rates were directly standardized by age (Association of Public Health Epidemiologists in Ontario, 2009) using the *Revised European Standard Population* (RESP) published in 2013 (Eurostat, 2013) to make comparisons possible.

### 2.2 Procedures and statistical analysis

Suicide rates are expressed per 100,000 population per year using the annual mid-year population estimates for the relevant year. Age-standardized suicide rates (ASSRs) in Hungary during the study period were calculated to make suicide rates comparable over time.

Male-to-female ratios were calculated as the ratio of the incidence rate for males relative to that for females.

Our first approach to estimating incidence rate ratios (IRRs) and their 95% confidence intervals (CIs) was to employ Poisson regression models. However, the assumptions of this model were violated; therefore, the NB regression model was used for overdispersed count data (Agresti, 2003) to investigate the effect of possible risk factors (gender, age group, region, marital status and educational attainment) in relation to dying by suicide.

Therefore, the NB regression model was used for overdispersed count data to investigate the effect of possible risk factors (gender, age group, region, marital status and educational attainment) in relation to dying by suicide.

Seasonal variation in suicide deaths was investigated using NB regression in analyses overall, by gender and suicide method first: month of death was the only independent factor included in the model. Moreover, sine and cosine terms were included in the model to control for annual seasonality. Thereafter, one of the significant risk factors (obtained from the “initial” NB regression noted before) was added to the (fundamental) model as an explanatory variable.

All these models mentioned above included the corresponding stratum-specific population (broken down by levels of a single given risk factor) as an exposure variable.

There are other methods for analyzing cyclical trends (Rau, 2007). In addition to NB regression, we used the geometrical model introduced by Edwards (Edwards, 1961), where the appropriate at-risk population could not be assigned to the suicide deaths. Consequently, data on the month of death were aggregated over the study period, and cyclical trends (seasonal peaks) in these monthly data were investigated using the methods noted above.

The quarters were defined as Q1 (January, February and March), Q2 (April, May and June), Q3 (July, August and September) and Q4 (October, November and December).

Trends in the annual number of deaths overall and by gender were also investigated employing NB regression. Since monthly populations were being estimated, the Edwards method was used to confirm NB regression findings in a seasonal pattern.

*p*-values of less than 0.05 were considered statistically significant. All analyses were performed with STATA Software Version 9.0 (StataCorp LP, College Station, TX, USA).

## 3 Results

In total, 60,210 suicide deaths (45,753 males and 14,457 females; 76% and 24%, respectively) were registered in Hungary during the 1995–2017 period. The over 35s (the groups aged 35–49, 50–64 and over 65 years, combined) accounted for more than six-sevenths of all victims (51,793 cases, 86%).

Hanging, self-poisoning by drugs and jumping from a height were the most common (specified) methods of suicide, with 37,594 (62.4%), 7133 (11.8%) and 3864 (6.4%) deaths, respectively.

### 3.1 The pattern of suicide by sociodemographic factor

Table 1 displays summary statistics for suicide cases and suicide rates (expressed as deaths per 100,000 people at risk) in Hungary during the study period by sociodemographic subgroup.

**Table 1:** Descriptive statistics of suicide cases and crude suicide rates in Hungary during 1995–2017 by factor.

Subgroup	Population	Number of deaths	SR (95%CI)	1995–2017 SR change	Male-to-Female ratio (95%CI)
GENDER					
Male	110006527	45753	41.6 (41.2–42)	−47%	3.48 (3.42–3.55)
Female	121048933	14457	12 (11.8–12.2)	−54%	
AGE GROUP					
0–19 years	51144509	1075	2.1 (2–2.2)	−47%	3.49 (3.01–4.06)
20–34 years	49422180	7342	14.9 (14.5–15.2)	−48%	5.25 (4.92–5.59)
35–49 years	49339387	17234	34.9 (34.4–35.5)	−67%	4.33 (4.18–4.48)
50–64 years	44316074	17467	39.4 (38.8–40)	−46%	3.87 (3.75–4)
above-65 years	36833310	17092	46.1 (45.4–46.8)	−55%	3.33 (3.23–3.44)
STATISTICAL (NUTS2) REGION					
Central Hungary	66711903	14378	21.6 (21.2–21.9)	−48%	2.55 (2.47–2.65)
Central Transdanubia	25277988	5720	22.6 (22–23.2)	−41%	3.86 (3.63–4.12)
Western Transdanubia	22864409	4186	18.3 (17.8–18.9)	−35%	4.3 (3.98–4.64)
Southern Transdanubia	22086841	5392	24.4 (23.8–25.1)	−51%	3.66 (3.43–3.9)
Northern Hungary	28566295	7258	25.4 (24.8–26)	−57%	4 (3.78–4.24)
Northern Great Plain	34926322	11553	33.1 (32.5–33.7)	−52%	3.84 (3.68–4.02)
Southern Great Plain	30621702	11225	36.7 (36–37.3)	−50%	3.72 (3.56–3.89)
MARITAL STATUS (WHOLE POPULATION)					
Married	94759906	24791	26.2 (25.8–26.5)	−56%	4.34 (4.2–4.48)
Not married	136295554	35375	26 (25.7–26.2)	−46%	3.06 (2.99–3.14)
MARITAL STATUS (ABOVE-20 AGE GROUP)					
Married	94634867	24783	26.2 (25.8–26.5)	−56%	4.34 (4.2–4.48)
Not married	85276084	34274	40.2 (39.8–40.7)	−60%	3.44 (3.36–3.52)
EDUCATIONAL ATTAINMENT					
0-7 grades	5581765	2757	49.4 (47.6–51.2)	−38%	3.98 (3.66–4.32)
Primary School	42641140	16697	39.2 (38.6–39.8)	−32%	4.51 (4.35–4.68)
Vocational School	35930581	11399	31.7 (31.1–32.3)	−62%	4.82 (4.54–5.13)
Secondary Education	45724284	7723	16.9 (16.5–17.3)	−37%	3.38 (3.22–3.56)
Higher Education	23638192	2691	11.4 (11–11.8)	−41%	2.91 (2.68–3.17)

Abbreviations: SR Suicide Rate, 95%CI 95% Confidence Intervals.

#### 3.1.1 Gender

The crude numbers and rates are displayed in Table 1. The suicide rate is about three-and-a-half times higher among men than among women (41.6 versus 12 per 100,000 persons per year).

#### 3.1.2 Age group

The male-to-female ratio was the highest in the group aged 20–34 years (5.25), then this ratio declined progressively towards the older age groups, reaching a minimum of 3.33 in the group aged over 65 years.

#### 3.1.3 Age-specific rates

The age group with the largest number of suicides was different (Table 2): the group aged between 50 and 64 years (29% of all cases) overall, the 35-49-year-old age group (30.5%) for males and the group aged over 65 years (39.9%) for females, respectively. However, the highest age-specific suicide rates were observed in the latter age group both overall and by gender. Both in the whole population and by gender, growing rates were detected across age groups.

**Table 2:** Numbers of suicide deaths and suicide rates per 100,000 persons in 1995, 2017 and during 1995–2017.

*Age group*	1995		2017		1995–2017	
	Number of deaths	Age-spec. mort. rate	Number of deaths	Age-spec. mort. rate	Number of deaths	Age-spec. mort. rate
OVERALL						
*0–19*	82	3.05	31	1.62	1075	2,12
*20–34*	414	19.89	189	10.39	7342	14,89
*35–49*	1065	46.66	355	15.53	17234	34,93
*50–64*	821	46.98	493	25.4	17467	39,41
*65-*	984	68.18	565	30.70	17092	46,14
*Total*	3366	32.91	1633	16.68	60210	26,09
ASSR (95% CI)	36.69 (35.45–37.93)		16.5 (15.7–17.2)		27.27 (27.05–27.49)	
MALE						
*0–19*	73	5.31	21	2.14	844	3,25
*20–34*	338	31.90	170	18.19	6206	24,67
*35–49*	859	76.21	277	24.05	13971	56,94
*50–64*	591	74.38	363	39.79	13413	65,68
*65-*	614	112.83	409	58.90	11319	82,50
*Total*	2475	50.57	1240	26.53	45753	41,64
ASSR (95% CI)	59.69 (57.34–62.04)		28.2 (26.63–29.77)		46.2 (45.77–46.62)	
FEMALE						
*0–19*	9	0.69	10	1.07	231	0,93
*20–34*	76	7.44	19	2.15	1136	4,70
*35–49*	206	17.83	78	6.88	3263	13,16
*50–64*	230	24.13	130	12.64	4054	16,97
*65-*	370	41.16	156	13.62	5773	24,75
*Total*	891	16.70	393	7.68	14457	11,95
ASSR (95% CI)	18.06 (16.87–19.24)		7.21 (6.49–7.92)		11.99 (11.8–12.19)	

During the 1995–2017 period, all these age-specific rates fell (by slightly different extent: 47–67%). Similar drops can be observed gender-wise, with one exception: the rate for women aged under 20 years rose (from 0.69 to 1.07 per 100,000 persons, meaning an increase of 57%). In view of crude numbers, this represents only one additional victim in 2017 compared to nine suicide cases in 1995 (the corresponding population at risk declined by about 30%). However, it is worth noting that the number of suicide deaths for men in the same age group fell from 73 to 21 over the course of these 23 years. As a result, while there were eight times as many male victims as female ones in the age group below 20 years in 1995, there were barely more than twice as many in 2017.

#### 3.1.4 Age-standardized rates

In the total population, the ASSR between 1995 and 2017 was 27.27 per 100,000 persons per year (95% CI: 27.05–27.49). In the male and female subpopulations, the ASSRs during the same period were 46.2 (45.77–46.62) and 11.99 (11.8–12.19), respectively.

In 1995, the annual suicide rate was 36.69 per 100,000 persons (95% CI: 35.45–37.93); in 2017, the same rate was less than half of that: 16.5 (15.7–17.3). During the 23 years of the study, these rates were similarly halved for both genders: there was a decrease for males from 59.69 (57.3 4–62.04) to 28.2 (26.63–29.77) and for females from 18.06 (16.87–19.24) to 7.21 (6.49–7.92).

#### 3.1.5 Region

Western Transdanubia had the lowest suicide rates, both in 1995 and 2017, as well as in the whole period (also by gender); similarly, the Southern Great Plain had the highest rates in every respect.

However, there were also major changes in the order of rates: while Central Hungary fell from second place in 1995 to fifth place in 2017, Northern Hungary rose from fifth place to second place in the same period.

The male-to-female ratio was the lowest in Central Hungary (2.55) and the highest in Western Transdanubia (4.3).

#### 3.1.6 Marital status

In the whole population, the suicide rate was nearly the same for married persons and non-married ones. This apparent contradiction is resolved by the fact that there are few married persons in the age group below 20 years (and very few of them die of suicide).

If only the subpopulation over the age of 20 was considered, the mortality rate for unmarried persons was more than 1.5 times higher (40.2 versus 26.2 per 100,000 persons per year).

These rates decreased to a similar extent (by 56–60%) between 1995 and 2017 in the two groups. The male-to-female ratio was about 25% higher in the married subpopulation (4.34 versus 3.06).

#### 3.1.7 Educational attainment

During the 20-year period under examination, 1998–2017, suicide rates gradually decreased with higher levels of education. This was also the case for 2017; however, in 1998, primary school non-completers were not far behind vocational school graduates in this respect.

In 19 years, the suicide rate for the latter group dropped to nearly one-third (which was by far the biggest drop among the groups).

The largest male-to-female ratio was also observed in this group (4.82); after that, this ratio fell progressively towards those with higher educational attainment, reaching its minimum (2.91) in the most educated group (people with a college/university degree).

### 3.2 Risk factors

Table 3 shows incidence rates for groups within categories (compared to the group with the lowest risk of suicide in the category i.e. the *reference group*) obtained from the NB regression.

**Table 3:** IRRs for suicide during 1995–2017 by single risk factor using negative binomial regression.

Subgroup	OVERALL		MALE		FEMALE	
	Incidence Rate Ratio (95% CI)	p-value for risk factor	Incidence Rate Ratio (95% CI)	p-value for risk factor	Incidence Rate Ratio (95% CI)	p-value for risk factor
AGE GROUP						
0–19 years	1	p < 0.001	1	p < 0.001	1	p < 0.001
20–34 years	7.56 (6.94–8.23)		8.31 (7.54–9.16)		4.97 (4.24–5.82)	
35–49 years	18.27 (16.74–19.94)		19.34 (17.52–21.36)		15.8 (13.62–18.34)	
50–64 years	20.7 (19.09–22.45)		22.32 (20.33–24.5)		20.9 (18.09–24.19)	
above-65 years	23.71 (21.76–25.83)		27.87 (25.32–30.68)		30 (25.81–34.89)	
STATISTICAL (NUTS2) REGION						
West. Transdan.	1	p < 0.001	1	p < 0.001	1	p < 0.001
Central Hungary	1.18 (1.12–1.24)		1.05 (1–1.11)		1.93 (1.76–2.12)	
Central Transdan.	1.24 (1.18–1.3)		1.21 (1.14–1.27)		1.43 (1.29–1.59)	
South. Transdan.	1.33 (1.26–1.4)		1.3 (1.22–1.38)		1.63 (1.47–1.81)	
North. Hungary	1.39 (1.32–1.46)		1.38 (1.3–1.46)		1.6 (1.45–1.77)	
North. Gr. Plain	1.81 (1.72–1.9)		1.77 (1.67–1.87)		2.16 (1.96–2.38)	
South. Gr. Plain	2 (1.91–2.11)		1.96 (1.85–2.07)		2.46 (2.24–2.71)	
MARITAL STATUS (WHOLE POPULATION)						
Not married	1	p = 0.702	1	p = 0.042	1.35 (1.28–1.43)	p < 0.001
Married	1.01 (0.97–1.05)		1.05 (1–1.09)		1	
MARITAL STATUS (ABOVE-20 AGE GROUP)						
Married	1	p < 0.001	1	p < 0.001	1	p < 0.001
Not married	1.54 (1.47–1.62)		1.59 (1.51–1.67)		2 (1.88–2.12)	
EDUCATIONAL ATTAINMENT						
Higher Educ.	1	p < 0.001	1	p < 0.001	1	p < 0.001
Secondary Educ.	1.55 (1.47–1.64)		1.7 (1.6–1.81)		1.49 (1.36–1.63)	
Vocat. School	2.97 (2.81–3.13)		2.7 (2.54–2.87)		1.59 (1.43–1.76)	
Primary School	3.67 (3.47–3.88)		4.27 (4–4.55)		2.75 (2.51–3.01)	
0-7 grades	4.29 (3.96–4.65)		5.12 (4.66–5.62)		4.03 (3.58–4.54)	

Abbreviations: *IRR* Incidence Rate Ratio, *95%CI* 95% Confidence Intervals.

The risk of suicide was significantly (*p* < 0.001) higher in men than in women: a nearly three-and-a-half times larger incidence rate was detected during the study period (IRR = 3.48).

An increasing suicide risk was observed across age groups: the incidence rate (compared to the age group below 20 years) for groups aged 20–34 years, 35–49 years, 50–64 years and over 65 years was 7.56 (95% CI: 6.94–8.23), 18.27 (16.74–19.94), 20.7 (19.09–22.45) and 23.71 (21.76–25.83), respectively.

Western Transdanubia had the lowest risk of suicide among the regions. The incidence rate ratios (incidence rate compared to that region) in the next four regions (in this respect) were between 1.18 and 1.39; however, the suicide risk in the two worst regions was about twice as high (the Northern and Southern Great Plain: IRR = 1.81 and IRR = 2).

In the whole population, there was no significant difference (*p* = 0.714) between incidence rates for married persons and non-married ones; however, for the subpopulation aged over 20 years only, the suicide risk was significantly (*p* < 0.001) higher for married persons than for non-married ones (IRR = 1.54). We also examined the suicide risk for unmarried persons in the subpopulation aged over 15 years, which showed a slight decrease (IRR: 1.35, 95% CI: 1.29–1.41; *p* < 0.001).

A significant growing trend in suicide risk was detected among those with lower educational attainment: the incidence rate (compared to the most educated group i.e. people with a college/university degree) for people completing secondary, vocational and primary school, and primary school drop-outs was 1.55 (95% CI: 1.47–1.64), 2.97 (2.81–3.13), 3.67 (3.47–3.88) and 4.29 (3.96–4.65), respectively.

### 3.3 General trends

**Fig. 2:**
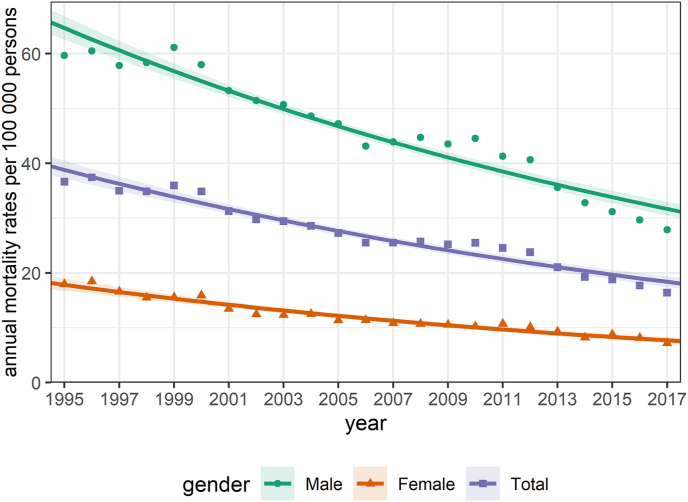
Yearly suicide trends in Hungary during 1995–2017 by gender. Annual suicide rates per 100,000 persons: observed (points) and fitted rates (solid lines) with confidence intervals (bands) obtained from negative binomial regression.

The NB regression model for annual data (Fig. 2) revealed a declining trend in the yearly suicide rates (IRR: 0.972, 95% CI: 0.969–0.975; *p* < 0.001). A similar significant (*p* < 0.001) decreasing trend was detected both for males (IRR: 0.972, 95% CI: 0.969–0.976) and females (IRR: 0.969, 95% CI: 0.966–0.972).

Using the Edwards test, a significant (*p* < 0.001) cyclical trend was found in monthly suicide deaths with a peak at the end of June. There was a similar significant (*p* < 0.001) seasonal pattern in suicide rates with a peak in June for both sexes, and there was essentially no gender difference in the peak (the difference was only a week). Seasonal investigations on a quarterly basis led to similar results: a significant (*p* < 0.001) peak at the end of Q2 (both overall and by gender).

### 3.4 Seasonality by risk factor

Table 4 displays seasonal trends in Hungary by subgroup during the 23 years of the study.

**Table 4:** Seasonal (monthly) suicide trends by levels of a single factor in Hungary during 1995–2017.

Subgroup	Seasonal peak	Angle	p-value	Amplitude	Goodness of Fit
GENDER					
Female	late-June	177.1	<0.001	0.149	0.424
Male	mid-June	169.8	<0.001	0.169	0.4
AGE GROUP					
0–19 years	–	–	0.202	–	–
20–34 years	late-June	173.6	<0.001	0.087	0.305
35–49 years	early-June	157.7	<0.001	0.149	0.355
50–64 years	late-June	171.4	<0.001	0.167	0.371
above-65 years	early-July	180.1	<0.001	0.226	0.429
STATISTICAL (NUTS2) REGION					
Western Transdanubia	late-June	173.5	<0.001	0.182	0.285
Central Hungary	mid-June	162.2	<0.001	0.107	0.433
Central Transdanubia	late-June	179.4	<0.001	0.163	0.339
Southern Transdanubia	early-June	155.4	<0.001	0.158	0.409
Northern Hungary	late-June	179	<0.001	0.178	0.308
Northern Great Plain	late-June	172.3	<0.001	0.205	0.38
Southern Great Plain	late-June	173.5	<0.001	0.191	0.39
MARITAL STATUS (WHOLE POPULATION)					
Not married	late-June	175.1	<0.001	0.155	0.42
Married	mid-June	166.9	<0.001	0.178	0.368
MARITAL STATUS (ABOVE-20 AGE GROUP)					
Married	mid-June	166.9	<0.001	0.178	0.368
Not married	late-June	175.2	<0.001	0.158	0.389
EDUCATIONAL ATTAINMENT					
Higher Education	late-June	171	0.002	0.098	0.317
Secondary Education	mid-June	163.5	<0.001	0.115	0.427
Vocational School	mid-June	164.7	<0.001	0.146	0.377
Primary School	late-June	172.6	<0.001	0.172	0.343
0-7 grades	mid-June	168.6	<0.001	0.289	0.458

Adding one of the significant risk factors to sine and cosine terms in the NB model, both the seasonal pattern and the factor remained significant. At each level of each factor, the number of suicide deaths reached its peak between early June and early July. Generally, no more than a half-month difference was detected in peaks in suicides within the levels of a given factor, except the region, for which the difference in peaks was almost a month. Goodness-of-fit (GoF) statistics for all models were tabulated.

### 3.5 Seasonality by suicide method

**Fig. 3:**
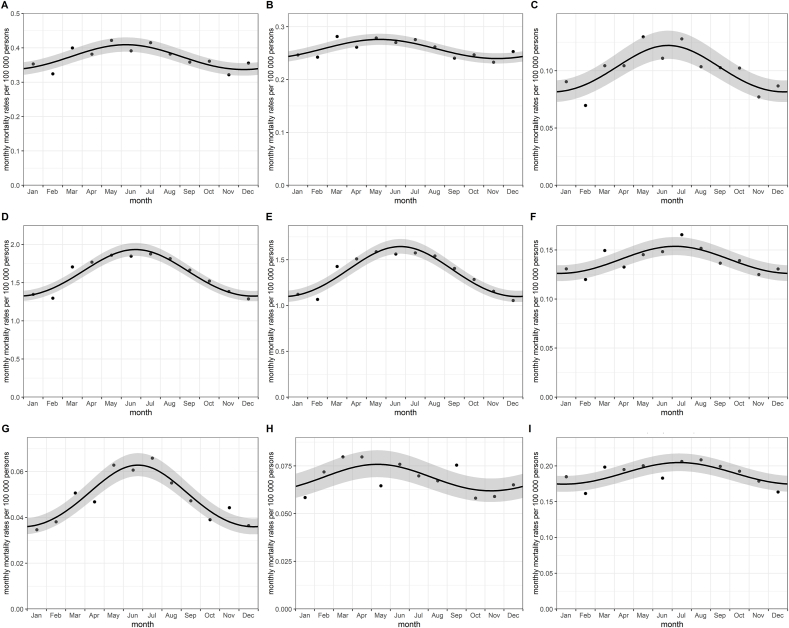
Seasonal (monthly) suicide trends in Hungary during 1995–2017 by suicide method. A. NON-VIOLENT methods (combined): p < 0.001 B. Self-poisoning by drugs: p < 0.001 C. Self-poisoning by chemicals and other substances: p < 0.001 D. VIOLENT methods (combined): p < 0.001 E. Hanging: p < 0.001 F. Jumping from a height: p < 0.001 G. Drowning: p < 0.001 H. Firearm: p = 0.007 I. OTHER (unspecified) methods: p < 0.001. Monthly suicide rates per 100,000 persons: observed (points) and fitted rates (solid lines) with confidence intervals (bands) obtained from negative binomial regression.

There were large differences in peaks by suicide method, as shown in Fig. 3.

The peak of non-violent suicides was in early June; suicides committed by violent methods peaked half a month later, in late June. However, within violent and non-violent methods, there were differences of more than one month (for firearm and jumping from a height) and almost two months (for drug overdose and chemical poisoning), respectively. Furthermore, as regards all the suicide methods, a difference of more than two months was also detected. Quarterly peaks were nearly the same as those observed in a monthly breakdown.

## 4 Discussion

### 4.1 Strengths and limitations

The data were obtained from vital registers; although Hungary's vital statistics performance index is one of the best in the world (Phillips et al., 2014), there may because-of-death biases (e.g. youth suicides registered as fatal traffic accidents). Additionally, the official Hungarian population estimates did not account for international migration in the period 1995–2001, which caused a sudden increase in population between 1999 and 2000. Despite the limitations of the published data, the trends and risk factors for suicide were calculated to present population-level processes as we believe that these are “negligible” at a population level for such a long period.

This epidemiological study on nationwide data relates to the risk of suicide by sociodemographic characteristic, such as gender, age group, region, marital status and educational attainment. Instead of only reporting suicide mortality rates (or ASSRs), we used regression models to estimate trends in suicide deaths in Hungary between 1995 and 2017. Overdispersion did not influence our results, as the NB regression method was employed in both risk estimation and investigation of the annual mortality trend. We also used the Edwards test in the monthly cyclical trend analyses, which confirmed the findings of the NB regression model, as both yielded similar results. Furthermore, the longest study period available (from a public database) was used in these seasonality analyses. A description of incidence rates in terms of seasonal variation is important in many epidemiological studies, as it could lead to preventive measures. As far as we are aware, this is the first epidemiological study to investigate the seasonal variation of suicide deaths by NUTS2 region in such a detailed way in Hungary.

### 4.2 Main findings

Risk estimates of suicide rates were calculated by gender, age group, region, marital status and educational attainment, highlighting a nearly three-and-a-half times greater risk in males than in females overall. The risk of suicide in males was more than threefold in all age groups. A significantly increased risk of suicide mortality was observed for non-married persons compared with married ones; moreover, greater age and lower education also raised the risk of suicide.

Significantly decreasing annual trends in suicide rates were found during the study period: the yearly suicide mortality rates halved in Hungary between 1995 and 2017. Similar trends were observed in both genders. The greatest percentage drop among all subgroups was seen in the group with middle educational attainment (people graduating from vocational school).

Significant seasonality was detected in monthly suicide mortality rates (both overall and by gender), with a peak in late June. The pattern of seasonal effect remained almost unchanged after adjusting the (sociodemographic) factors noted above for seasonality in NB regression models.

Seasonal trends with a peak from late June to mid-July were observed in mortality rates for all suicide methods except for firearm and drug overdose (mid- and late May, respectively). However, there was no significant seasonal variation in monthly suicide rates from gas poisoning, mostly due to the low number of cases.

In summary, our study describes the risk factors and pattern of trends in mortality rates for suicide in Hungary during the 1995–2017 period.

### 4.3 Comparison with other studies

Seasonal trends in suicide mortality rates were investigated in several studies. A similar peak in June was reported by Zonda and his colleagues (Zonda, 2005). In our study, risk factors were included in the models that investigated seasonal variation in suicide deaths; each of these factors remained significant (and, conversely, seasonality remained significant in the presence of each factor). Consequently, our study only proves that seasonal effects have been independently increasing the risk of suicide in early summer.

Environmental effects are involved in the etiology of mortality. The available Hungarian monthly mean temperature (OMSZ, 2010a), precipitation (OMSZ, 2010b) and sunshine duration (OMSZ, 2010c) data generally supported these hypotheses for suicide mortality. Unfortunately, there were no monthly (detailed) environmental data available to investigate causality more precisely. It is assumed that sunlight triggers suicidality via melatonin (Petridou et al., 2002) and serotonin transmission (Vyssoki et al., 2012). As regards seasonal rhythms, the duration of sunshine still shows a significant correlation with the frequency of suicide, but the extent of this effect is low (Vyssoki et al., 2014). Müller and his colleagues concluded that higher environmental temperature and global radiation correlate with increased suicidality (Muller et al., 2011).

In Hungary, Törő and her colleagues (Toro et al., 2009) found that the number of suicides increased during warm weather with low relative humidity (WorldData, 2020). A recent Hungarian study demonstrated that daily sunshine duration had a significant, immediate positive relationship with daily suicide rates (Bozsonyi et al., 2020).

Gender as a risk factor for suicide was investigated by various regression methods in several countries (Cubbin et al., 2000; Denney et al., 2009; Partonen et al., 2004), including Hungary (Balint et al., 2016); there was a 2.5- to fivefold suicide risk for males. In line with these studies, we also found a high (approximately 3.5-fold) suicide risk for men.

In agreement with studies conducted in different countries (Bjorksten et al., 2009; Kalediene et al., 2006; Sun et al., 2011), we also observed a significant seasonal pattern in suicide deaths with a peak in June for both genders. Similar results were reported by Zonda and his colleagues (Zonda, 2005) for a three-decade study period (1970–2000) in Hungary and by Sebestyén and her colleagues (Sebestyen et al., 2010) for a shorter, subsequent one in that country (1998–2006). There was no autumn rise in suicide deaths for women in Hungary, as indicated by some researchers (Hakko et al., 1998; Meares et al., 1981; Micciolo et al., 1989) in the literature. Some authors found different seasonal peaks in other countries (Nakaji et al., 2004; Rock et al., 2003).

Several studies (Ajdacic-Gross et al., 2006; Shah & De, 1998; Stack, 2000) that investigated the suicide risk of age came to the conclusion also reflected by our findings: the risk of suicide increases with age. By contrast, in England and Wales the male suicide rate was the highest in the 25–34-year-old age group (McClure, 2000).

Many studies have reported a relationship between age and seasonal pattern in suicide (Hakko et al., 1998; Maes et al., 1993; Preti & Miotto, 1998; Sun et al., 2011); however, their findings (in terms of peaks) were quite different. According to Woo and his colleagues (Woo et al., 2012), the inconsistency between these studies might reflect methodological and environmental differences. We observed that seasonal variation was more pronounced among older age groups, a finding which is in agreement with some of the studies referenced above (Christodoulou et al., 2009; Hakko et al., 1998; Preti & Miotto, 1998).

Rihmer and his colleagues presented higher suicide rates in south-eastern Hungary compared with those in the north-western parts of the country (Rihmer et al., 2013). Another Hungarian research group found an elevated suicide risk for both men and women living on the Great Plain (in the east and south-east) and stated that this effect cannot be explained by education, marital status or other demographic factors (Balint et al., 2019).

In agreement with the studies mentioned above, we observed a significant (almost twofold) suicide risk for the regions on the Great Plain (compared to Western Transdanubia, the region with the lowest risk of suicide).

The seasonal variation of suicide deaths by NUTS2 region was similar. We found that even the maximum distance of these peaks was less than a month (early vs. late June).

Like other studies (Almasi et al., 2009; Balint et al., 2016; Lorant, Kunst, Huisman, Bopp, et al., 2005; Park et al., 2018), we also found that marriage was a protective factor against suicide, although the suicide risk for non-married persons was slightly lower (about 1.5-fold) than those reported in the studies noted above.

The seasonal variation in suicides was investigated by marital status (single, married, widowed or divorced) in Finland (Nayha, 1983). This study by Näyhä found a seasonal peak in spring or early summer for all categories of marital status (in the case of both genders), but also a second peak in autumn for married and widowed females.

By contrast, we considered marital status as a binary variable (married or non-married); the difference between the peaks (both in June) was less than half a month.

As regards education, we concluded that higher levels of educational attainment are accompanied by a lower risk of suicide; these are consistent with the findings of several previous studies (Balint et al., 2016; Lorant et al., 2018; Park et al., 2018). However, the relationship between educational attainment and suicide risk is less obvious for women (Balint et al., 2016; Lorant, Kunst, Huisman, Costa, et al., 2005).

As far as we are aware, there is no study to date that has published the seasonal pattern of suicides by educational attainment; however, our study revealed that there was almost no difference between the peaks (mid- or late June) in Hungary.

It is already known that there is a seasonal variation in suicide deaths committed by violent methods (Ajdacic-Gross et al., 2005; Bjorksten et al., 2009; Christodoulou et al., 2009; Hakko et al., 1998; Kalediene et al., 2006; Lin et al., 2008; Maes et al., 1993; Preti & Miotto, 1998; Rasanen et al., 2002; Toro et al., 2009). All these studies reported a seasonal peak in spring or early summer, which is consistent with the general pattern of suicide mortality. As compared with our study, some of them (Ajdacic-Gross et al., 2005; Bjorksten et al., 2009; Preti & Miotto, 1998) revealed a peak in June. As elsewhere, hanging was the main violent method in Hungary; like other studies (Ajdacic-Gross et al., 2005; Christodoulou et al., 2009; Preti & Miotto, 1998), we noted a seasonal peak in June for these suicide deaths.

In contrast to certain studies (Kalediene et al., 2006; Lin et al., 2008; Maes et al., 1993; Preti & Miotto, 1998), we found a seasonal pattern for non-violent suicide methods with a peak in June. However, we also observed that violent methods of suicide had a more marked seasonal variation compared with non-violent ones.

As far as we know, this is the first study in Hungary to investigate the seasonality of suicide by method at a population level.

## 5 Conclusions

Several sociodemographic risk factors and (both annual and seasonal) patterns of suicide deaths were investigated in this ecological study using well-established statistical methods. Despite the declining trends in annual mortality rates for suicide, the Hungarian suicide rate is still high. To our knowledge, this has been the first study to investigate the seasonal pattern so extensively in Hungary. In line with the general seasonal peak in late June, similar peaks (between early June and early July) were observed within the levels of each risk factor and the seasonality remained significant even in the presence of any of the factors; this has confirmed the significant role of environmental effects in the etiology of suicide mortality.

These findings may prove useful in developing preventive strategies (e.g. cultural education programs on suicide and its seasonal pattern conducted with the involvement of the media – targeting both the civilian population and health professionals – to pay more attention to men, the elderly and patients with depressive disorder, especially in summer).

## Ethics approval

No ethics approval was necessary according to the Hungarian Law in usage of statistical data (Act XLVI of 1993, Sec. 10).

## Funding

This research was funded by the 10.13039/501100000780European Union and the State of Hungary, co-financed by the European Social Fund within the framework of EFOP-3.6.1-16-2016-00008. The funder had no final role in the study design; in the collection, analysis and interpretation of data; in the writing of the article; or in the decision to submit the paper for publication.

## CRediT authorship contribution statement

**Tamás Lantos:** Conceptualization, Methodology, Software, Formal analysis, Investigation, Data curation, Writing – original draft, Writing – review & editing, Visualization. **Richard J.Q. McNally:** Methodology, Formal analysis, Writing – original draft, Writing – review & editing, Supervision. **Tibor András Nyári:** Conceptualization, Methodology, Formal analysis, Data curation, Writing – original draft, Writing – review & editing, Supervision.

## Declaration of competing interest

none.
